# Seroprevalence of Infection with Feline Morbilliviruses Is Associated with FLUTD and Increased Blood Creatinine Concentrations in Domestic Cats

**DOI:** 10.3390/v13040578

**Published:** 2021-03-30

**Authors:** Johannes Busch, Romy M. Heilmann, Thomas W. Vahlenkamp, Michael Sieg

**Affiliations:** 1 Institute of Virology, Faculty of Veterinary Medicine, Leipzig University, An den Tierkliniken 29, 04103 Leipzig, Germany; Johannes.Busch@vetmed.uni-leipzig.de (J.B.); Vahlenkamp@vetmed.uni-leipzig.de (T.W.V.); 2 Department for Small Animals, Faculty of Veterinary Medicine, Leipzig University, An den Tierkliniken 23, 04103 Leipzig, Germany; romy.heilmann@kleintierklinik.uni-leipzig.de

**Keywords:** feline morbillivirus, paramyxovirus, seroprevalence, blood creatinine, chronic kidney disease, FLUTD, immunofluorescence assay

## Abstract

Feline morbilliviruses (FeMV) are fairly newly discovered paramyxoviruses found in cats. The first description indicated an association with widely distributed chronic kidney disease (CKD) in the host species. In various studies, a global prevalence and a further genotype, designated FeMV-2, and the involvement of other organ systems in infected individuals were shown. Using an immunofluorescence assay, we detected an overall seroprevalence of FeMV in almost half of the cats investigated (*n* = 380), with a significantly increased proportion in younger animals. In comparison to European Shorthair cats, the rate of seropositivity is higher in pedigree cats. Regardless of the breed, FeMV infection was associated with increased blood creatinine concentrations, suggesting an association with CKD. Further analysis indicated that this association was the strongest in animals having high IFA titers against FeMV-2. In addition, a significant association between FeMV-positive status and the prevalence of feline lower urinary tract disease (FLUTD, or idiopathic cystitis) was detected. This association was dominated by cats having antibodies against FeMV-1 only. To further evaluate the positive correlation between FeMV seroprevalence and CKD as well as FLUTD, consideration of additional clinical characteristics and laboratory parameters is warranted, and controlled infection studies with both FeMV genotypes are necessary. Clinicians should, however, be aware of a possible link between renal and lower urinary tract disease and FeMV infections.

## 1. Introduction

Companion animals are common in Germany, and with almost 15 million individuals living in 23% of the households in 2019, cats present the largest group [1,2]. A study revealed that up to 50% of randomly selected cats have evidence of chronic kidney disease (CKD) based on blood biochemistry profiles, urinalysis, and diagnostic imaging, while not showing acute (or any) signs of illness [3]. The pathological manifestation of CKD is often characterized by tubulointerstitial inflammation and renal fibrosis [4,5] concomitant with a progressive loss of renal function. An acute deterioration of CKD (ACKD) is frequently associated with anorexia, lethargy, vomiting, and weight loss [6]. Surveillance of cats for CKD is of great clinical importance because severe tissue damage and loss of organ function have occurred once clinical signs are noticed. A reduced glomerular filtration rate can be detected by measuring urine specific gravity (USG), and glomerular damage can cause proteinuria. Furthermore, the blood creatinine concentration can help to evaluate renal function but is not a good estimator of the glomerular filtration rate (GFR) in cats and dogs [7]. Early diagnosis of CKD is important for initiating successful therapeutic interventions [8]. For this purpose, the International Renal Interest Society (IRIS) established a staging method involving serum creatinine and symmetric dimethylarginine (SDMA) as most useful prognostic markers for CKD in domestic cats. In addition, a combination of physical examinations combined with hematology, biochemistry, urine analysis, and blood pressure measurements should be initiated in senior cats once they reached an age between seven and ten years [9]. Based on these guidelines, nutritional restriction of phosphorous is most effective in prolonging the survival time of cats with CKD [10]. In addition to increased age, ischemic events, metabolic disorders, endocrinopathies, infectious diseases, and primary inflammatory conditions might contribute to the onset and progression of CKD reviewed in [11]. In this review, bacteria, parasites, and viruses, including feline leukemia virus (FeLV), feline foamy virus (FFV), feline immunodeficiency virus (FIV), and feline morbillivirus, were assessed as potential causative agents.

Feline idiopathic cystitis commonly referred to as feline lower urinary tract disease (FLUTD), is a common and often recurring condition affecting the urinary bladder and urethra [12]. Particularly male cats with this condition are often presented with urethral obstruction, which is considered a medical emergency [13]. Several patient (e.g., obesity) and environmental factors (e.g., high levels of stress) are known to increase the risk of FLUTD episodes [14]. Few viruses, including feline calicivirus, have been evaluated for a possible link with FLUTD pathogenesis [15], but the etiologic role of viruses in FLUTD is currently unknown and remains to be further investigated.

Feline morbilliviruses (FeMV, formerly known as FmoPV) were first detected in stray cats from Hong Kong. Viral RNA was found primarily in urine samples rather than in rectal swaps or blood, and a significant association with tubulointerstitial nephritis (TIN) was proposed [16]. The latter was supported by the immunohistochemically detected colocalization of FeMV and histopathological lesions within the renal parenchyma [17]. Other investigations could not find a link between FeMV infection and renal dysfunction [18,19]. FeMV RNA was detected mainly in urine samples and tissues lining the urinary tract obtained from cats by research groups in Japan [20], Germany [21], Italy [22,23,24,25,26], USA [27], Brazil [28,29,30], Turkey [31], UK [18], Malaysia [19], and mainland China [32], with prevalence ranging from 2.5% to 50.8%. With this increasing evidence for a potential role of FeMV in feline urinary tract pathologies, more insight into the diversity of the viral strains became obvious [24,33,34]. Phylogenetical surveillance led to the detection of distinct subtypes (A to D) within the FeMV-1 cluster [25,34]. In 2019, a new genotype, designated FeMV-2, was reported, displaying a nucleotide homology of 78% to the formerly known FeMV-1 [35]. The seroprevalence of FeMV-specific antibodies in cats was investigated using single protein-based assays and revealed positive rates of 18.8%–30% [16,18,24,34,36]. Using an immunoblot, 22% of investigated feline sera were positive for FeMV antibodies [33], while 63% of cats were positive by an immunofluorescence-based assay (IFA) in another study [37]. Considering the diversity of FeMV, the method of detection thus appears to be important to avoid underestimating the prevalence of FeMV in the feline population.

In this double-blind study, sera from a large number of cats were collected in a tertiary veterinary care facility in central Germany and were screened for FeMV antibodies via IFA using FeMV-1 as well as FeMV-2 infected cells. For each animal, the antibody titer against either genotype was determined, and the seroprevalence was calculated. In addition, serological data were tested for the possibility of a correlation with the blood creatinine concentration as a marker of renal insufficiency and for an association with the cats’ diagnoses.

## 2. Materials and Methods

### 2.1. Study Population and Sample Collection

Feline sera (*n* = 840) were obtained from the Department for Small Animals, Veterinary Teaching Hospital, Faculty of Veterinary Medicine, Leipzig University, Germany. These surplus materials were from serum samples collected between 2013 and 2017 from feline patients during routine clinical diagnostic investigations and/or follow-up evaluations during treatment, all unrelated to this study. The Department for Small Animals at Leipzig University uses a standard consent form for patient admission and treatment, which allows for the use of residual serum samples for research projects. Sera were analyzed by immunofluorescence assay with the investigators (J.B., M.S.) blinded to any clinical patient data to avoid a potential bias. Patient data were extracted from the medical records.

### 2.2. Virus Stock Preparation

LLC-MK2 cells and CrFK cells were used for the propagation of FeMV-GT2 (“Gordon” strain) [35] and of FeMV-GT1 (“Lapön” strain) [38], respectively. Cells were grown in 75 cm^2^ cell culture flasks to reach 80%–90% confluence. Infection was performed using a MOI of 0.1 in 4 mL Dulbecco’s Modified Eagle Medium (DMEM) (Gibco, Waltham, MA, USA). After incubation for 2 h at 37 °C, 90% humidity and 5% CO_2_, the virus-containing inoculum was removed and replaced with 10 mL DMEM containing 2% (*v*/*v*) FBS (Sigma, Germany), 1.0 mM sodium pyruvate, non-essential amino acids, and 100 IU/mL penicillin-streptomycin (Gibco, Waltham, MA, USA). Cells were cultured for seven days at the indicated conditions. Hereafter, the cell culture supernatants were centrifuged at 500× *g* at 4 °C for 5 min to remove any debris. Virus titration was performed using the endpoint dilution assay in the cell line used for propagation, followed by immunofluorescence staining using a primary antibody against the nucleoprotein of feline morbillivirus (rabbit polyclonal, produced in-house according to the methods described previously [18]) and AlexaFluor^®^488-labelled secondary antibody (Thermo Fisher Scientific, Waltham, MA, USA). Viral titers were expressed as 50% tissue culture infectious dose (TCID_50_).

### 2.3. Immunofluorescence Assay

Immunofluorescence assay was performed as described previously [37]. Briefly, CrFK cells were infected with FeMV-1 and LLC-MK2 cells with FeMV-2. Every other vertical row of wells was infected to provide an uninfected (MOCK) control cavity adjacent to an infected one. After five days, cells were fixed with ice-cold, 80% acetone (*v*/*v*) at –20 °C for 10 min. Non-specific binding sites were blocked with 5% (*w*/*v*) BSA in PBS for 30 min at 37 °C. Cat sera were diluted 1:100(*v*/*v*) in 1% BSA in PBS and incubated for 1 h at 37 °C or 14 h at 4 °C. Each serum was tested against both FeMV genotypes and the corresponding control of MOCK-infected cells. For visualization, an anti-cat AlexaFluor^®^488-conjugated secondary antibody was used and incubated for 30 min at 37 °C. Nuclei were counterstained using DAPI (Carl Roth, Karlsruhe, Germany). Sera from cats persistently infected with FeMV and serum samples from SPF cats served as positive and negative controls, respectively. Titration of positive sera was performed at 2-fold dilution steps starting at a 1:100 dilution. To account for interindividual bias during the evaluation of intensities, these analyses were independently conducted by two investigators (J.B., M.S.) that were blinded to the individual sera. For statistical analyses, very weak positive sera were considered negative because detected viral foci did not correlate with the morphology of infectious particles used for infection, nor could these be clearly distinguished from background fluorescence.

### 2.4. Blood Creatinine Determination

Blood creatinine concentrations (reference interval: 71–159 μmol/L) were measured using the ‘Fuji DRI-CHEM NX500 i’ chemistry analyzer (scil animal care company GmbH, Viernheim, Germany) according to the manufacturer’s instructions.

### 2.5. Statistical Analysis

Seroprevalence was calculated as the ratio of IFA positive samples to the total number of sera. For statistical analysis, only those sera were included from cats for which the parameters sex, age, breed, and blood creatinine concentration were available. To test for statistical significance, GraphPad Prism v9.0.0 (San Diego, CA, USA) and JMP v13.1.0 (SAS Institute, Cary, NC, USA), was used. Fisher’s exact test or a likelihood ratio test was performed to compare the proportions of individuals between or among the study groups. Wilcoxon’s signed-rank test was performed to compare matched values and Mann–Whitney’s test to compare unpaired values between two groups.

## 3. Results

### 3.1. Study Population

For serological analysis, 380 individual cats were screened. As most cats were hospitalized for several days at the Department for Small Animals, Leipzig, Germany, multiple serum samples were available from the same animals in most cases so that 840 samples were analyzed in total. This cohort included 66% male (77% neutered, 23% intact) and 34% female (82% spayed, 18% intact) cats. The study population had an overall even age distribution, with 29% of the cats up to five years of age, 35% between 5.5 and 10 years old, and 36% of the cats being older than 10 years. The predominant breed was the European Shorthair (ESH) (*n* = 256) and ESH crossbreeds (*n* = 4) (73%), while the remaining 27% were pedigree cats. Included in this group of cats were British shorthair (BSH, *n* = 17) and BSH crossbreed cats (*n* = 2), Britannica cats (*n* = 1), Birman cats (*n* = 4), Carthusian cats (*n* = 6) and one crossbreed, Maine coon cats (*n* = 11) and crossbreeds (*n* = 2), Norwegian forest cats (*n* = 7) and one crossbreed, Persian cats (*n* = 13) and crossbreeds (*n* = 8), Ragdoll cats (*n* = 3) and one crossbreed, Siamese cats (*n* = 3) and one crossbreed, Siberian cats (*n* = 2), Thai cats (*n* = 2), one Rex curly Shorthair, one Bengal cat, one Neva Masquerade cat, one Russian Blue cat, one Somali cat, and 27 cats of unspecified breeds.

A number of different diseases affecting different organ systems were diagnosed in the study population (for one cat the final diagnosis was not documented). A total of 165 cats (43%) had a disease affecting the urinary tract, while 83 cats (22%) were diagnosed with an episode of FLUTD.

### 3.2. FeMV Seroprevalence

**Figure 1:**
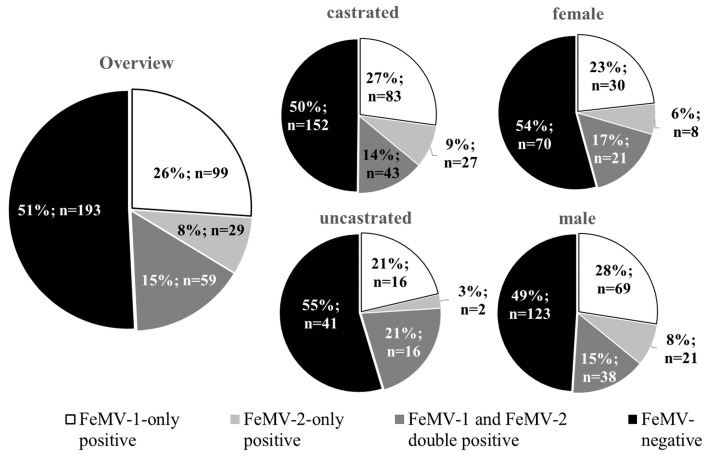
Percentages of FeMV-1‑only, FeMV-2‑only, as well as FeMV-1 and FeMV-2 double-positive feline sera. Seroprevalence is displayed as an overview including all animals (*n* = 380; left panel) and also stratified by sex and castration status (right panels).

To determine the prevalence of FeMV infection in the geographic area of central Germany, the presence of antibodies against both FeMV genotypes wase evaluated. The data analyses revealed that 26% (*n* = 99) of the cats included in this study were serologically positive for FeMV-1. In addition, 8% (*n* = 29) of the cats were seropositive for FeMV-2 and 15% (*n* = 59) for both FeMV-1 and FeMV-2. Neither sex nor castration status were correlated with the presence or absence of FeMV antibodies (Figure 1).

**Figure 2:**
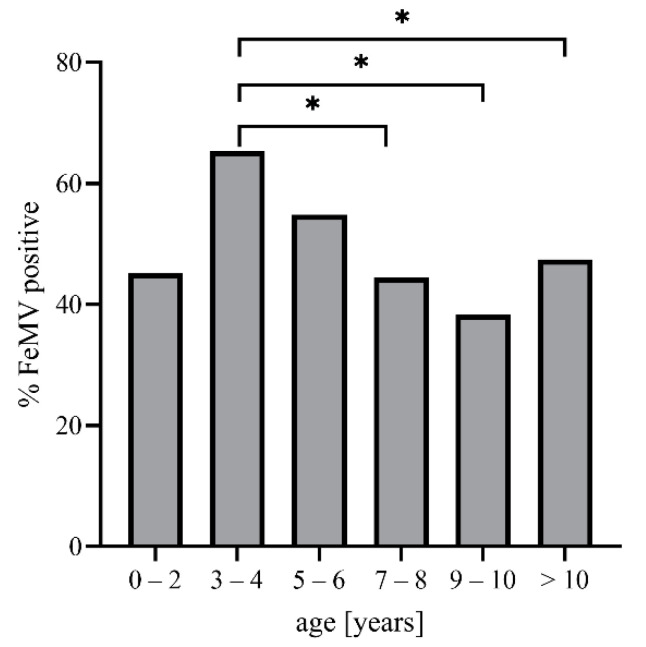
Correlation between the detection of FeMV antibodies in feline sera and the age of the animals. The percentage of seropositivity was calculated for each age group. *p*-values were determined using Fisher´s exact test: * *p* ≤ 0.05.

To further investigate the overall seroprevalence of 49%, potential associations with the animals’ age and breed were assessed. The analysis revealed a significantly higher overall seroprevalence in cats that were 3–4 years old (65%) compared to animals that were seven years of age or older (7–8 years: 44%, *p* = 0.0474; 9–10 years: 38%, *p* = 0.0138; and >10 years: 47%, *p* = 0.0451). No significant difference in the antibody status was seen between any of those age groups and cats up to two years of age (45% seropositive; Figure 2).

**Figure 3:**
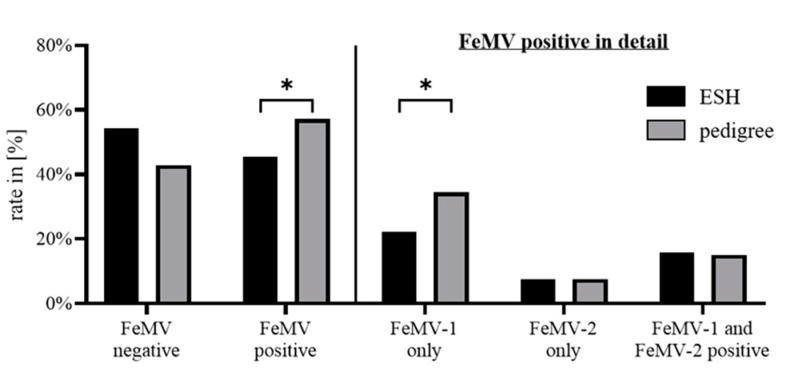
Association between FeMV antibody status and the cats’ breed. FeMV positivity was evaluated in general and for the particular antibody species detected. *p*-values were determined using Fisher´s exact test. * *p* ≤ 0.05.

Evaluating for a link between cat breed and antibody status revealed that 56% of pedigree cats are serologically FeMV-positive, whereas only 46% of ESH cats are (*p* = 0.0163). This significant difference is caused by a higher rate of FeMV-1 specific antibodies in pedigree cats (34%) compared to ESH cats (22%). Breed-dependent effects were not detected with FeMV-2-only or double-positive status (Figure 3).

There was no association between FeMV antibody status and the presence of other infectious diseases (7% in FeMV-negative group vs. 6% in FeMV-positive group, *p* = 0.890). The following infectious etiologies were detected in the FeMV-negative group: FeLV (*n* = 2), FIP (*n* = 1), feline herpesvirus-1 (*n* = 1), feline panleukopenia (*n* = 1), suspected infection/fever of unknown origin (*n* = 8). Similar frequencies were observed in the FeMV-positive group: FIV (*n* = 2), FIP (*n* = 1), feline herpesvirus-1 (*n* = 3), feline calicivirus (*n* = 3), mycoplasma sp. (*n* = 3), feline panleukopenia (*n* = 2), lungworm (*n* = 1), suspected infection/fever of unknown origin (*n* = 3). There was also no significant difference for infectious diseases among the three FeMV-positive subgroups (*p* = 0.079).

### 3.3. Correlation of Blood Creatinine Values with FeMV-Antibody Responses

**Figure 4:**
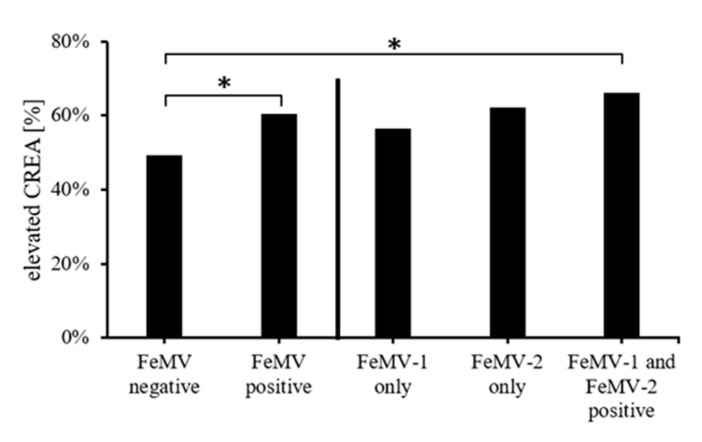
Association between FeMV seropositivity and increased blood creatinine levels (≥159 µmol/L). FeMV positivity was evaluated in general and for the particular antibody specificity detected. P-values were determined using Fisher´s exact test. * *p* ≤ 0.05.

Due to the FeMV seroprevalence being very high in the population of cats investigated, possible clinical consequences of these infections were further studied. Of all individual cats analyzed, 55% (*n* = 208) had increased blood creatinine concentrations (>159 µmol/L). Within the group of FeMV-seronegative cats, the percentages of animals with increased vs. normal creatinine levels were almost equal (49% and 51%). Of the FeMV-seropositive animals, 60% (*n* = 113) had increased creatinine levels while 74 animals (40%) had creatinine concentrations within the reference values. Association testing revealed a significantly higher rate of azotemic cats (i.e., animals with increased blood creatinine levels) in the FeMV-positive group than in the FeMV-negative one (*p* = 0.0307). This association was predominantly caused by the FeMV-1 and FeMV-2 double-positive group (Figure 4).

**Figure 5:**
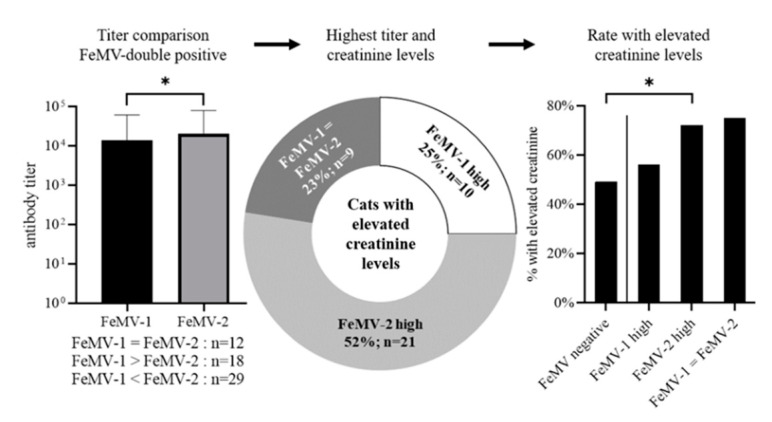
The group of FeMV-double-positive cats was further investigated. Antibody titers against each viral genotype were determined (left) and evaluated for a correlation with the blood creatine levels (middle). Furthermore, proportions of animals with increased creatinine levels were compared to the FeMV-negative group (right). * *p* ≤ 0.05.

Next, antibody titers against both FeMV-1 and FeMV-2 were further evaluated for any potential association with the increased blood creatinine levels in the double-positive group of cats. On average, the titres against FeMV-2 were significantly higher (mean = 20,159) than those titres against FeMV-1 (mean = 14,105; *p* = 0.0256). Within the FeMV-double-positive group, 18 cats had higher titers for FeMV-1 than FeMV-2 (labeled ‘FeMV-1 high’), 29 cats had higher ertiters for FeMV-2 than FeMV-1 (labeled ‘FeMV-2 high’), and 12 cats exhibited equal titers against both FeMV genotypes (Figure 5, left panel).

Of all 59 FeMV-double positive animals, 40 (68%) had increased creatinine levels in the blood. A closer evaluation of these 40 animals showed that 52% (*n* = 21) belonged to the ‘FeMV-2 high’ group, whereas 25% (*n* = 10) were ‘FeMV-1 high’ and 23% (*n* = 9) had equal titres against the two viruses (Figure 5, middle panel). Comparing these proportions of animals with increased blood creatinine concentrations in the three groups of FeMV-double-positive animals to those proportions in the FeMV-negative group revealed that increased blood creatinine levels were significantly more frequent only in the ‘FeMV-2 high’ group (*p* = 0.0270; Figure 5, right panel).

### 3.4. Association of FeMV-Antibody Response with Clinical Diagnoses

To further investigate the possible link between FeMV infections and diseases of the urinary tract, the proportions of cats with urinary tract diseases (including urolithiasis, neoplasia, CKD, acute renal failure, FLUTD) between the groups of cats were compared and no significant difference found (*p* = 0.172) between FeMV-negative (77/192 = 40%) and FeMV-positive (88 in 187 = 47%) animals. There were also no significant differences (p = 0.482) in the prevalence of urinary tract diseases among the three FeMV-positive groups: FeMV-1-only (49 in 99 = 50%), FeMV-2-only (15 in 29 = 52%) and FeMV-1/2-double (24 in 59 = 41%). Similar results were observed for cats with and without confirmed and staged CKD. Prevalence of CKD in the FeMV-negative group (37 in 192 = 19%) and the FeMV-positive group (30 in 187 = 16%) was found to be not significantly different (*p* = 0.410). Accordingly, for confirmed CKD there was no significant difference (*p* = 0.137) among the three FeMV-positive groups: FeMV-1-only (11 in 99 = 11%), FeMV-2-only (seven in 29 = 24%) and FeMV-1/2-double (12 in 59 = 20%).

**Figure 6:**
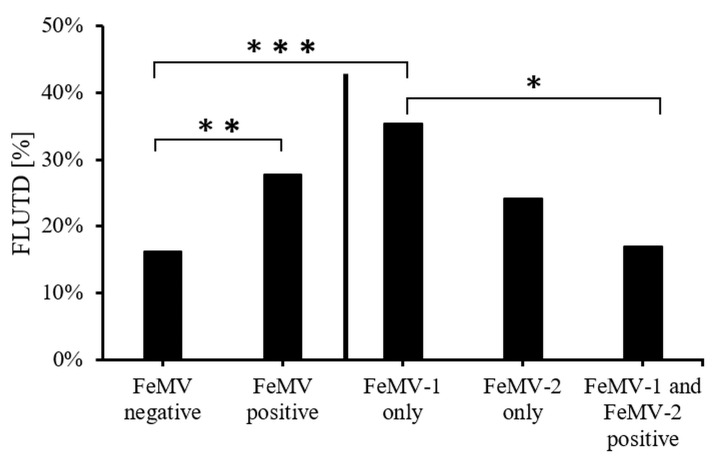
Association between FeMV seropositivity and FLUTD prevalence. FeMV positivity was evaluated in general and for the particular antibody specificity detected. P-values were determined using Fisher´s exact test. *p* ≤ 0.05 = *, *p* ≤ 0.01 = **, *p* ≤ 0.001 = ***.

In contrast, a significantly (*p* = 0.006) higher portion of cats with FLUTD was found in the FeMV-positive group (52 in 187 = 28%) when compared to the FeMV-negative group (31 in 192 = 16%). Further, this positive association was mainly determined by the differences of FLUTD prevalence between the FeMV-1-only (35 in 99 = 35%) and the FeMV-negative group (*p* = 0.0003). Differences in FLUTD diagnosis between the FeMV-2-only (seven in 29 = 24%) vs. FeMV-negative (31 in 192 = 16%) and FeMV-1/2-double (10 in 59 = 17%) vs. FeMV-negative were not significant (*p* = 0.295 and *p* = 0.800, respectively). However, a significant difference (*p* = 0.0174) was found in the FLUTD prevalence between the FeMV-1-only and the FeMV-1/2-double group (Figure 6).

## 4. Discussion

The worldwide prevalence and diversity of FeMV mostly rely on the detection of virus-specific RNA in urine or tissue specimens. Only a few studies with small numbers of samples have been conducted to date to investigate the serological response to these viruses in felines. Here we describe the FeMV-seroprevalence in a large study including domestic cats from a large geographic area in central Germany. We found a high overall prevalence of antibodies against FeMV (45%) which is higher than the 18.8%–30% seroprevalence of FeMV-specific antibodies reported from cats in Hong Kong [16], Japan [34,36], the UK [18], and Italy [24]. The higher seroprevalence in our study may be due to the differences in the methodologies used as previous studies were based on single protein (N or P protein of FeMV) assays (ELISA, western blot, or IFA on transfected HeLa cells). In contrast, we used an IFA based on FeMV-infected cells allowing for the detection of serological reactions against all structural and non-structural viral proteins. The performance of this established and validated assay was reported in a study including free-roaming domestic cats in Chile, where a high FeMV seroprevalence of 63% was detected [37]. Previous results using whole virus Western blot analysis of feline serum samples revealed varying reaction patterns against the N, P, M, H, and L proteins of FeMV [33]. Therefore, serologic assays based on single viral proteins may underestimate the true infection rate. In addition, all previous assays were developed to detect FeMV-1 proteins only. In 2019, we described a new genotype of FeMV, tentatively designated as FeMV-2, with an amino acid diversity of structural proteins between these two genotypes of 75–92% [35]. Interestingly, serological reactions directed against either FeMV genotype were found in individual feline serum samples in the current study. This finding was also described in the Chilean group of domestic cats [37]. Both observations reflect the diversity of antigenic epitopes between FeMV-1 and FeMV-2 and point towards the presence of non-cross-reactive antibodies against the two FeMV genotypes.

No association between FeMV prevalence and sex or castration status of the cats was observed in this study. However, it needs to be emphasized that approximately 75% of the cats included in this study were neutered, rendering the statistical power to detect a possible association of FeMV prevalence and castration rather low. A significantly higher FeMV prevalence was detected in cats aged 3–4 years compared to older animals. A similar peak in younger cats was also observed for feline leukemia virus (FeLV) infections [39]. Young age was also positively correlated with infection rates of feline coronavirus (FCoV) [40]. The higher FeMV-seroprevalence observed in cats less than 4 years of age might reflect the more frequent social interactions between cats, increased roaming distance, and also sexual activity in this age group. A significantly higher proportion of FeMV-1 positive cats was observed in pedigree cats when compared to ESH cats. This phenomenon may be explained by the genetic bottleneck in pedigree cats resulting in an increased risk of genetic predispositions to viral infections (or infections in general) compared to cats with a broader genetic background, as has been proposed for FCoV infections [40]. However, this effect was significant only in the ‘FeMV-1 only’ group of cats. Given the lack of data from controlled infection experiments in domestic cats, such a mechanistic background appears to be a reasonable explanation for the breed-related association detected, but this hypothesis requires further investigation. An alternative explanation could be that FeMV-1 is the predominant genotype circulating in certain feline breeds.

The initial discovery of FeMV was related to the detection of tubulointerstitial nephritis (TIN) on histopathological analysis of a small number of selected cats [16]. Following this first description, several research groups have aimed to estimate the role of FeMV in feline acute and/or chronic kidney disease, in part with incongruent results [18,19]. Here we further investigated the link between FeMV seroprevalence and azotemia (i.e., increased blood creatinine concentrations), a widely accepted marker for renal insufficiency. We found a significantly higher proportion of FeMV-double-positive cats with increased serum creatinine concentrations compared to FeMV-negative animals. Assuming blood creatinine levels to reflect renal insufficiency or stable disease states, the proportion of FeMV-double-positive cats with marked azotemia or advanced disease (67.8%) was twice that detected of non-azotemic FeMV-double-positive cats (32.2%). ‘FeMV-1 only’ and ‘FeMV-2 only’ sera were not detected to have significantly increased blood creatinine concentrations. One limitation of this study is that only animals presenting at a university small animal clinic were investigated, rendering healthy cats to be underrepresented. This confounder is also reflected by detecting an increased blood creatinine concentration in 55% of all animals included in this study.

Evaluating the nature of FeMV-double-positive serum samples included titrating these sera for both FeMV genotypes. With regard to the antibody titers detected, our observations are consistent with the FeMV-1 seroprevalences reported from Japan [34] and Italy [24]. Interestingly, in this double-positive group of cats, more than half of the animals showed higher titer against FeMV-2 (designated as ‘FeMV-2 high’). These cats were also the major contributor to the significantly increased blood creatinine concentrations when compared to the FeMV-negative group. Sera with higher titres against FeMV-1 (designated as ‘FeMV-1 high’) or equivocal titres against both genotypes did not include significantly increased blood creatinine levels. This IFA staining pattern points to a differential immunological response to FeMV in individual animals, which warrants further study. The extent of FeMV titers did not allow for definitive conclusions as to the infection of individual cats in the double-positive group with a particular genotype, the reason being that two- or four-fold differences in titers made it difficult to address a specific genotype. On the other hand, ‘FeMV-1 only’ and ‘FeMV-2 only’ sera most likely reflect a prior infection with the respective FeMV genotype that led to a genotype-specific humoral immune response. Therefore, it cannot be concluded that the observed association with increased blood creatinine levels is genotype-specific. One explanation for the detection of double-positive sera could be that these reflect consecutive infections with both FeMV genotypes. Alternatively, these could also result from a re-stimulation with the same genotype followed by a booster in the humoral immune response leading to higher titers with the risk of cross-reactivity in the IFA.

In addition to increased serum creatinine concentrations, we showed that FeMV-seroprevalence is significantly associated with episodes of FLUTD and that this association is primarily driven by the higher FLUTD prevalence in the FeMV-1-only group of cats. Therefore, it appears reasonable to speculate that a genotype-related difference exists in the pathogenesis of FeMV. Due to the initial link of FeMV with TIN [16], nearly all consecutive research efforts have focused on renal pathology. Although some studies also included clinical diagnoses such as polyuria/dysuria [41] and other urinary tract disorders [42], these analyses were based on PCR-detection of FeMV and cannot be compared to our serological results. Moreover, histopathological data showed that the urothelium and the lamina propria mucosa of the urinary bladder are susceptible to FeMV-1 as proven by immunohistochemistry [24]. Thus, further investigation of the link between FeMV infection and FLUTD in cats, particularly those cats with repeated episodes requiring hospitalization and potentially surgical intervention, is warranted.

Our observed association of increased blood creatinine levels and FLUTD with a distinct serologic reaction pattern may explain the discrepancies with the results of previous studies. For instance, a small study in the UK including 40 cats did not find an association between FeMV seropositivity and azotemia [18], but in this analysis, a Western blot of FeMV-1 N protein was used. Hence, serum samples with antibodies generated against other structural proteins of FeMV-1 and FeMV-2 may have been missed. Also, only older cats, 8.7 to 18.5 years old (median: 12.5 years), were included in this study. Another study from Malaysia investigated 27 cats, from which blood biochemistries were available, for the presence of FeMV-1 in urine samples and found 18 animals to be positive. Nine of these cats had increased blood urea and creatinine levels, whereas the remaining 10 cats were not azotemic [19]. In contrast, a study from Turkey found increased blood creatinine levels in three cats (age: two months, four years, and 12 years) that were positive for FeMV-1 RNA in the urine, but sero-epidemiological analyses were not performed [31]. Recently, an Italian group investigated 14 FeMV-infected cats, 21 animals with CKD, and 22 healthy cats and found comparable serum creatinine values among these three groups of cats. However, urine protein-to-creatinine ratios (UPC) were significantly increased in the FeMV and CKD group compared to healthy cats [41]. As a limitation, we could not evaluate urinary parameters in our study because urine samples or urinalysis results were available from only a minority of the 380 cats included in this investigation.

In summary, these results suggest a complex interaction between FeMV and feline renal pathophysiology, presenting a challenge to prove or disprove a causative role of FeMV in feline CKD. In addition, we showed that parts of the urinary tract other than the kidneys should also be considered as susceptible targets for FeMV infections. To further elucidate this role, additional larger double-blind clinical studies and experimental infection experiments are needed, which should include blood and urine chemistry parameters as well as other infectious and non-infectious agents [11]. Also, our study showed that differential immunological responses to FeMV might be involved in the pathogenesis of FeMV infections in cats.

## Data Availability

The data set analyzed for the current study is available from the corresponding author upon reasonable request.
